# Zika virus infection triggers caspase cleavage of STAT1

**DOI:** 10.1128/spectrum.03609-23

**Published:** 2023-11-29

**Authors:** Jun Shu, Xiao Ma, Jingyi Zou, Zhenghong Yuan, Zhigang Yi

**Affiliations:** 1 Key Laboratory of Medical Molecular Virology (MOE/NHC/CAMS), School of Basic Medical Sciences, and Shanghai Institute of Infectious Disease and Biosecurity, Fudan University, Shanghai, China; 2 Shanghai Public Health Clinical Center, Fudan University, Shanghai, China; Shandong First Medical University, Jinan, Shandong, China

**Keywords:** flavivirus, zika virus, interferon, cleavage, interferon antagonism, STAT1, caspase

## Abstract

Zika virus (ZIKV) is an important pathogen that causes Guillain-Barre syndrome in adults and congenital Zika syndrome in newborns. Interferon (IFN) triggers intracellular antiviral signaling through two essential mediators STAT1 and STAT2, which are critical for host innate antiviral immunity. ZIKV has been reported to antagonize host IFN signaling by diverse mechanisms, including NS5-dependent and -independent ablation of STAT2. Whether flaviviruses target STAT1 remains controversial. In this study, we used infectious clones of ZIKV MR766 as tools and found that ZIKV infection triggered caspase-dependent cleavage of STAT1 during late infection. Further studies identified that the cleavage site is the aspartic acid 694, and the D694A mutation rendered STAT1 resistant to ZIKV infection-induced cleavage. Murine STAT1 (mSTAT1) was resistant to cleavage in ZIKV-infected cells. Ectopically expressed STAT1.D694A and mSTAT1 exerted comparable anti-ZIKV activity upon IFN treatment, challenging the role of STAT1 cleavage in IFN antagonism. We further knocked out STAT1 in the mouse embryonic fibroblast NIH3T3 cells, and STAT1 knockout dramatically augmented ZIKV infection, suggesting a STAT1-mediated viral restriction. Intriguingly, in the STAT1-knockout NIH3T3 cells, complementation of mSTAT1.D695G, a cleavage-sensitive variant, and the wild-type mSTAT1 both restored comparable anti-viral activities. These data suggest that caspase-mediated STAT1 cleavage is dispensable for IFN antagonism in ZIKV-infected cells.

**IMPORTANCE:**

Zika virus (ZIKV) is a re-emerging flavivirus. Similar to other flaviviruses, ZIKV antagonizes the host interferon (IFN) signaling pathway to establish infection. Understanding the molecular mechanism by which ZIKV antagonizes IFN-induced antiviral signaling may lead to a new antiviral strategy by cracking the IFN antagonism. Flaviviruses have been reported to employ NS5-dependent and -independent mechanisms to block STAT2-mediated signaling, whereas whether flaviviruses target STAT1 remains controversial. Herein, we found that ZIKV infection triggered caspase-dependent cleavage of STAT1 at the aspartic acid 694 during late infection, whereas murine STAT1 (mSTAT1) was resistant to cleavage. Intriguingly, ectopically expressed cleavage-resistant human STAT1.D694A or complementation of cleavable mSTAT1.D695G exerted comparable anti-ZIKV activity with their counterparts, challenging the role of caspase-mediated STAT1 cleavage in the IFN antagonism in ZIKV-infected cells. These data may also imply a dominant role of the antagonism of STAT2 but not STAT1 in ZIKV-infected cells.

## INTRODUCTION

Zika virus (ZIKV) re-emerged several times in recent years and poses a global health threat (1 – 3). ZIKV infection causes Guillain-Barre syndrome in adults and microcephaly in infants (4 – 7). There is currently no specific drug or vaccine available for ZIKV. ZIKV is a family member of the flavivirus. The genome of ZIKV is a single positive-sense RNA that is composed of 10,794 nucleotides. The viral genome encodes an open reading frame (ORF) that is translated into a polypeptide. The polyprotein is cleaved into three structural proteins and seven non-structural proteins (8). Structural proteins C, prM/M, and E are components of virion. Non-structural proteins NS1, NS2A, NS2B, NS3, NS4A, NS4B, and NS5 are mainly responsible for viral replication and evasion of host innate immunity (9, 10).

Innate immunity plays an important role in the first line of host defense against viral infection (11). Viral infection activates the innate immune system of the host and induces interferon (IFN) production. IFN-α binds to their receptors (IFNARs) and activates the Janus kinases, resulting in tyrosine phosphorylation of IFNARs, which further recruits and phosphorylates STAT2 and STAT1 (12 – 14). Phosphorylated STAT1 and STAT2 bind to interferon regulatory factor 9 to form IFN-stimulated gene factor 3 (ISGF3) (15, 16). Upon translocation to the nucleus, ISGF3 binds to IFN-stimulated response element (ISRE) in the chromosome to activate the expressions of IFN-stimulated genes (ISGs) (17). These ISGs exert antiviral activity in many stages of the virus life cycle and prevent the establishment of virus infection (18, 19).

Flaviviruses adopt a variety of strategies to antagonize the host IFN signaling pathways by targeting STAT2 proteins, either in an NS5-dependent or NS5-independent manner. Dengue virus (DENV) NS5 induces the degradation of STAT2 (20). Yellow fever virus (YFV) NS5 binds to STAT2 to inhibit the binding of ISGF3 to ISRE and antagonize the IFN signaling (21). ZIKV NS5 binds to STAT2 and causes the degradation of STAT2 in a proteasomal-dependent manner (22, 23). Our previous studies revealed that ZIKV infection triggers the suppression of host *de novo* protein synthesis to accelerate the degradation of short-lived, ubiquitinated STAT2 (24).

The effect of ZIKV infection on STAT1 is controversial. Several studies reported that ZIKV does not affect STAT1 expression (22, 23). Kuo et al. reported that STAT1 knockout mice were highly sensitive to ZIKV (25), suggesting that STAT1 signaling plays an important role in restricting ZIKV infection. NS5 of the Japanese encephalitis virus (JEV) interferes with the phosphorylation of Tyk2 and STAT1 and inhibits the nuclear translocation of STAT1 (26). Tick-borne encephalitis virus (TBEV) NS4A blocks the phosphorylation of STAT1 and STAT2 and the dimerization of STAT1 and STAT2 (27). ZIKV infection reduces the phosphorylation of STAT1 and STAT2 (28, 29).

In this study, by using an infectious clone of ZIKV MR766 (C7) as a tool, we found that ZIKV infection triggered caspase-dependent cleavage of STAT1 at aspartic acid 694 during late infection. Murine STAT1 (mSTAT1) was resistant to ZIKV infection-induced cleavage and STAT1 knockout in mouse embryonic fibroblast NIH3T3 cells dramatically augmented ZIKV infection. Intriguingly, functional complementation experiment in the STAT1-knockout NIH3T3 cells revealed a dispensable role of caspase-mediated STAT1 cleavage in IFN antagonism in ZIKV-infected cells.

## RESULTS

### ZIKV infection induced a reduction of STAT1 protein levels

**Fig 1:**
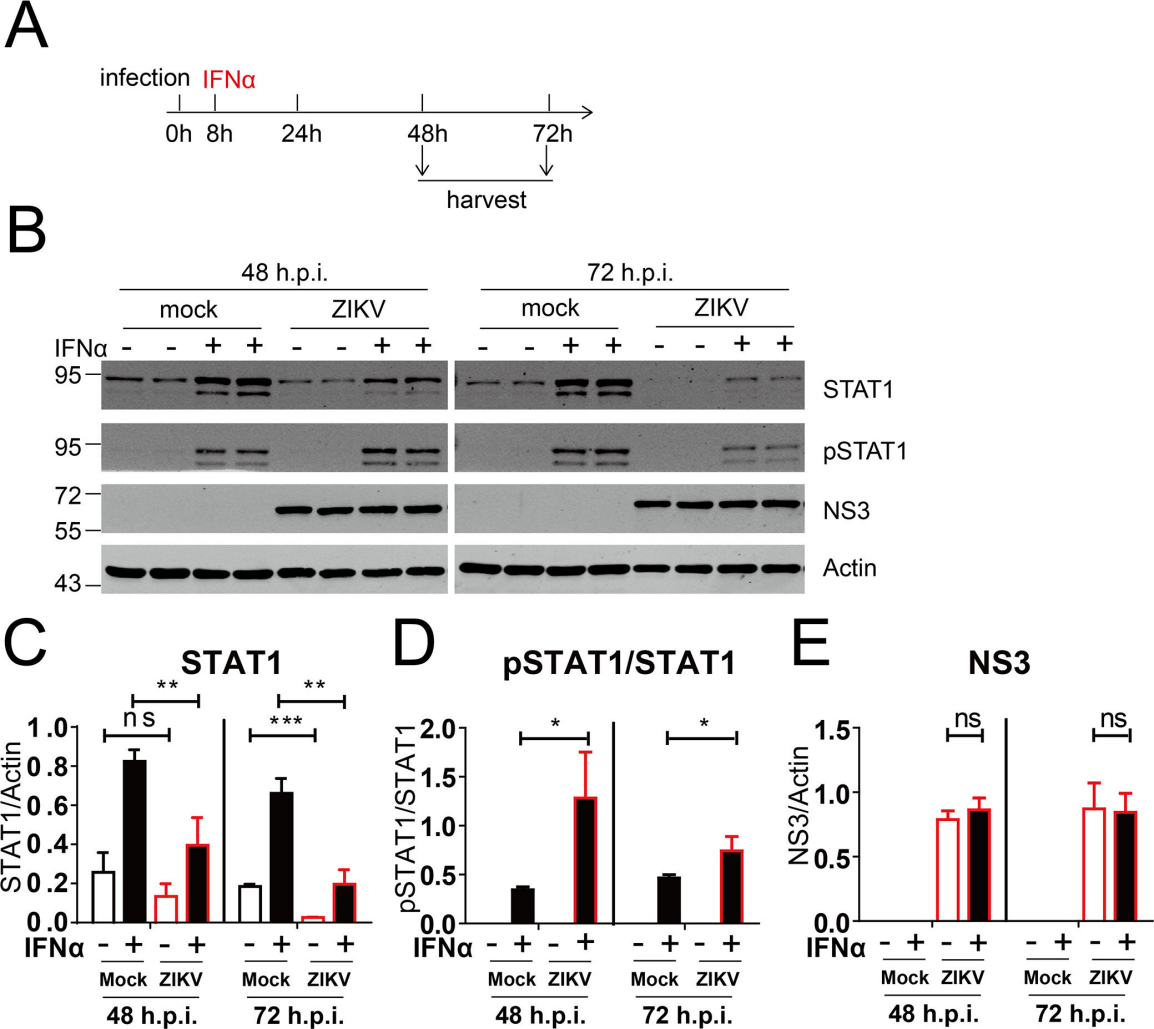
ZIKV infection induced a reduction in STAT1 protein levels. (**A**) Schematic representation of the experimental design. Huh7.5 cells were infected with ZIKV (MOI = 5) for 8 h and then treated with IFN-α (2,000 U/mL). Cells were harvested at 48 and 72 h post-infection. (**B**) The cells were analyzed by western blotting with indicated antibodies. A representative picture of three biological replicates is shown. The values to the left of the blots are molecular sizes in kilodaltons. (**C–E**) The protein abundances of each protein in B were quantified and plotted. The mean ± SD of three biological replicates is shown (*n* = 3). Statistical analysis was performed between the ZIKV-infected groups and the uninfected groups (ns, not significant; *P < 0.05; **P < 0.01; and ***P < 0.001; two-tailed, unpaired *t*-test).

It has been reported that phosphorylation of STAT1 was reduced in flavivirus-infected cells (26 – 29). We first examined the effect of ZIKV infection on the protein levels of STAT1 and phosphorylated STAT1 (pSTAT1). We infected Huh7.5 cells with ZIKV MR766 (C7) at an MOI of 5 and then treated the cells with 2,000 U/mL IFN-α. We analyzed the protein levels of STAT1 and p-STAT1 at 48 and 72 h after infection (Fig. 1A). ZIKV infection significantly reduced the protein levels of STAT1 in the absence or presence of IFN treatment at 48 and 72 h post-infection (Fig. 1B and C). After normalization (p-STAT1/total STAT1), the data showed that ZIKV infection did not affect the STAT1 phosphorylation (Fig. 1B and D). IFN treatment did not affect viral protein levels in the ZIKV-infected cells (Fig. 1B and E). Taken together, these results indicate that ZIKV infection reduces the protein levels of STAT1 but not the phosphorylated STAT1.

### ZIKV induced STAT1 cleavage

**Fig 2:**
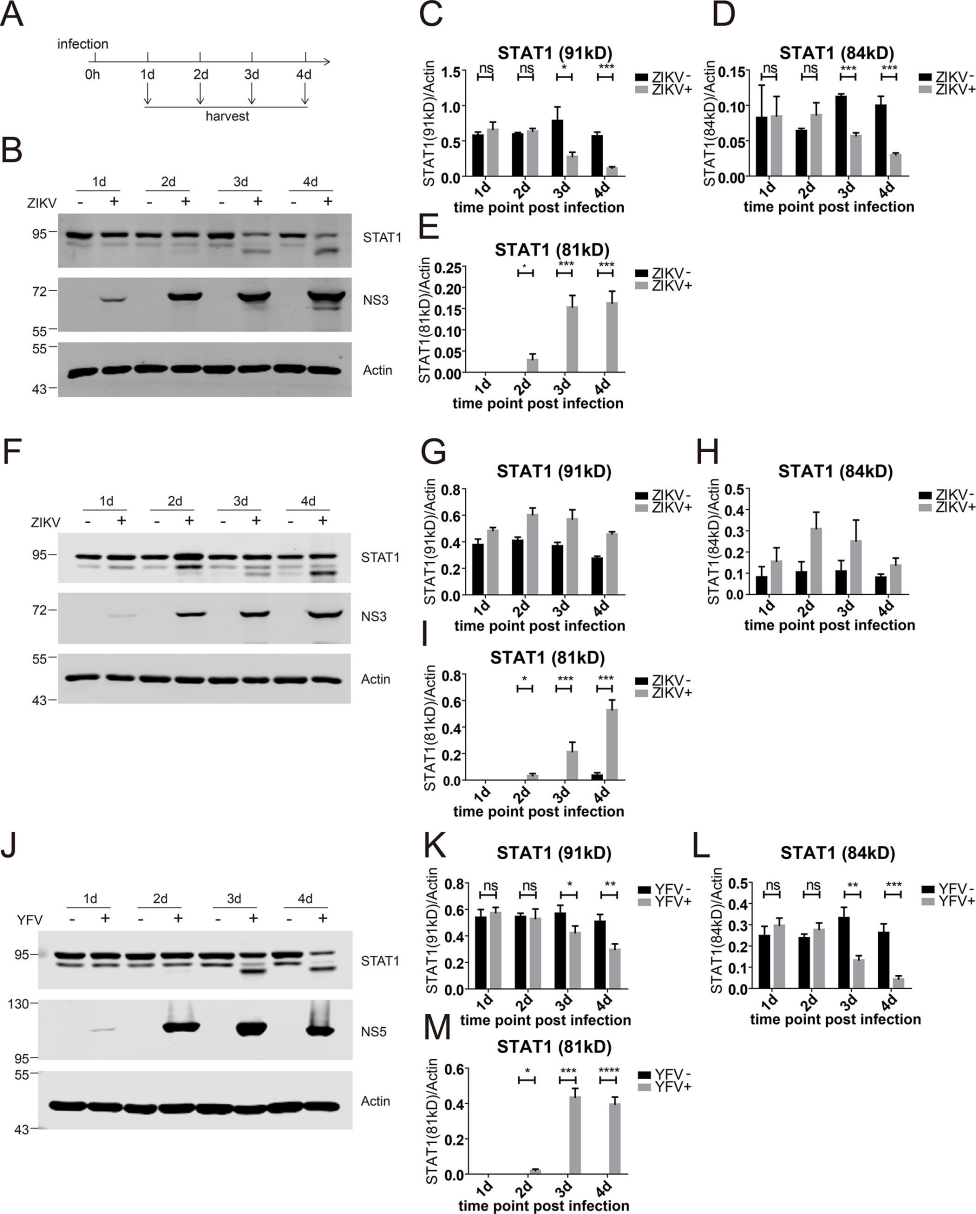
ZIKV and YFV induced STAT1 cleavage. (**A**) Schematic of the experimental design for panels B–E. Huh7.5 cells were infected (+) or not infected (−) with ZIKV (MOI = 1) and harvested at the indicated time points. (**B**) Western blotting analysis of Huh7.5 cells with the indicated antibodies. Representative pictures of three biological replicates are shown. The values to the left of the blots are molecular sizes in kilodaltons. (**C–E**) The protein abundances of each protein in panel B were quantified and plotted. The mean ± SD of three biological replicates is shown (*n* = 3). (**F**) SF268 cells were infected (+) or not infected (−) with ZIKV (MOI = 10) and harvested at the indicated time points. Western blotting analysis of the cells with the indicated antibodies. Representative pictures of three biological replicates are shown. The values to the left of the blots are molecular sizes in kilodaltons. (**G-I**) The protein abundances of each protein in panel F were quantified and plotted. The mean ± SD of three biological replicates is shown (*n* = 3). (**J**) Huh7.5 cells were infected (+) or not infected (−) with YFV (YF17D, MOI = 1) and harvested at the indicated time points. Western blotting analysis of the cells with the indicated antibodies. Representative pictures of three biological replicates are shown. The values to the left of the blots are molecular sizes in kilodaltons. (**K–M**) The protein abundances of each protein in panel J were quantified and plotted. The mean ± SD of three biological replicates is shown (*n* = 3). Statistical analysis was performed between YFV-infected groups and uninfected groups (ns, not significant; *P < 0.05; **P < 0.01; and ***P < 0.001; two-tailed, unpaired *t*-test).

We then infected Huh7.5 cells with ZIKV for various intervals and examined the protein expression of STAT1 more carefully. We observed that there were two species of STAT1, a major band with a molecular weight of 91 kDa and a minor band with a molecular weight of 84 kDa in the uninfected cells. From 3 days post-infection, the expression levels of the 91 kDa STAT1 and the 84 kDa STAT1 were significantly reduced in the infected cells (Fig. 2B through E). Strikingly, ZIKV infection resulted in the production of a new 81 kDa STAT1 species from 2 days post-infection (Fig. 2B and E). These results suggest that ZIKV infection affects the expression of STAT1 and probably leads to STAT1 cleavage during late infection. To further verify this observation, we infected human glioblastoma cell line SF268 with ZIKV for various intervals and examined the protein expression of STAT1. Consistently, ZIKV infection also resulted in the production of an 81 kDa STAT1 species in the ZIKV-infected SF268 cells (Fig. 2F through I). We also infected Huh7.5 cells with a YFV vaccine strain (17D) and examined the protein expression of STAT1. Consistent with ZIKV, YFV infection also resulted in the production of an 81 kDa STAT1 species in the infected cells (Fig. 2J through M). Taken together, these results indicate that flavivirus infection induces the production of the 81 kDa STAT1, which is probably a cleaved STAT1 species.

### ZIKV infection induced reduction of STAT1 in a proteasome-independent manner

**Fig 3:**
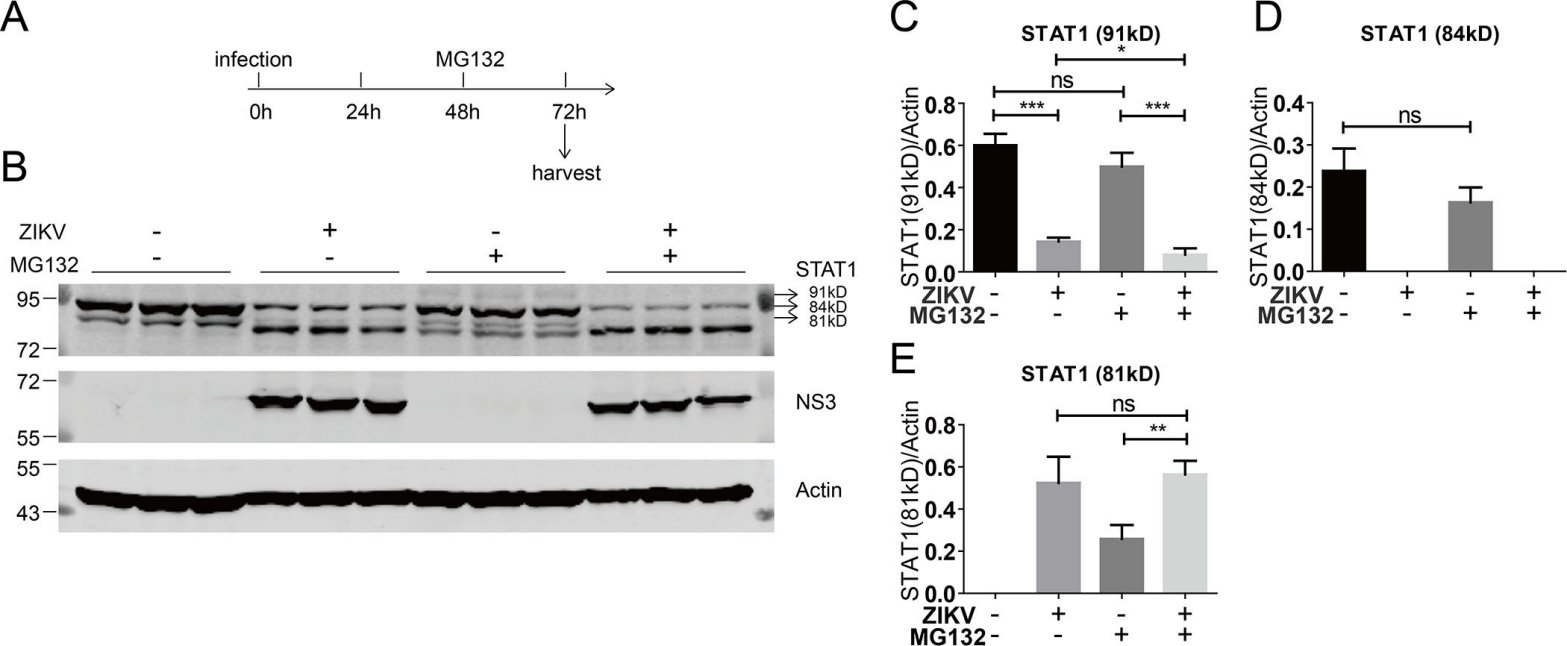
MG132 could not restore ZIKV infection-induced STAT1 cleavage. (**A**) Schematic of the experimental design. Huh7.5-GFPu cells in triplicates were infected with ZIKV (MOI = 1) for 48 h and then treated with MG132 (10 µM) for 24 h and harvested. (**B**) Western blotting analysis of the cells with the indicated antibodies. Representative pictures of three biological replicates are shown. The values to the left of the blots are molecular sizes in kilodaltons. (**C–E**) The protein abundances of each protein in B were quantified and plotted. The mean ± SD of three biological replicates is shown (*n* = 3). Statistical analysis was performed between indicated pairs (ns, not significant; *P < 0.05; **P < 0.01; and ***P < 0.001; two-tailed, unpaired *t*-test).

Previous studies have reported that ZIKV infection induces the degradation of human STAT2 in a proteasome-dependent manner (22, 23). To explore whether ZIKV infection affects the expression of STAT1 in a similar manner, we used the Huh7.5 cell line that stably expresses an unstable GFP (GFPu), which is used as a monitor of proteasome activity (30). When Huh7.5-GFPu cells were treated with proteasome inhibitor MG132, abundant GFPu was readily detected, indicating efficient inhibition of proteasomal activity (data not shown). We then examined if MG132 could restore STAT1 expression in ZIKV-infected cells. Huh7.5-GFPu cells were infected with ZIKV at an MOI of 1. At 2 days post-infection, the cells were treated with 10 µM MG132 for 24 h and then the protein level of STAT1 was analyzed (Fig. 3A). Obvious reduction of the full-length STAT1 and generation of the putative cleaved 81 kDa STAT1 were observed in the ZIKV-infected cells, but MG132 did not restore the STAT1 expression (Fig. 3B through E).

### ZIKV infection induced caspase-dependent cleavage of STAT1

**Fig 4:**
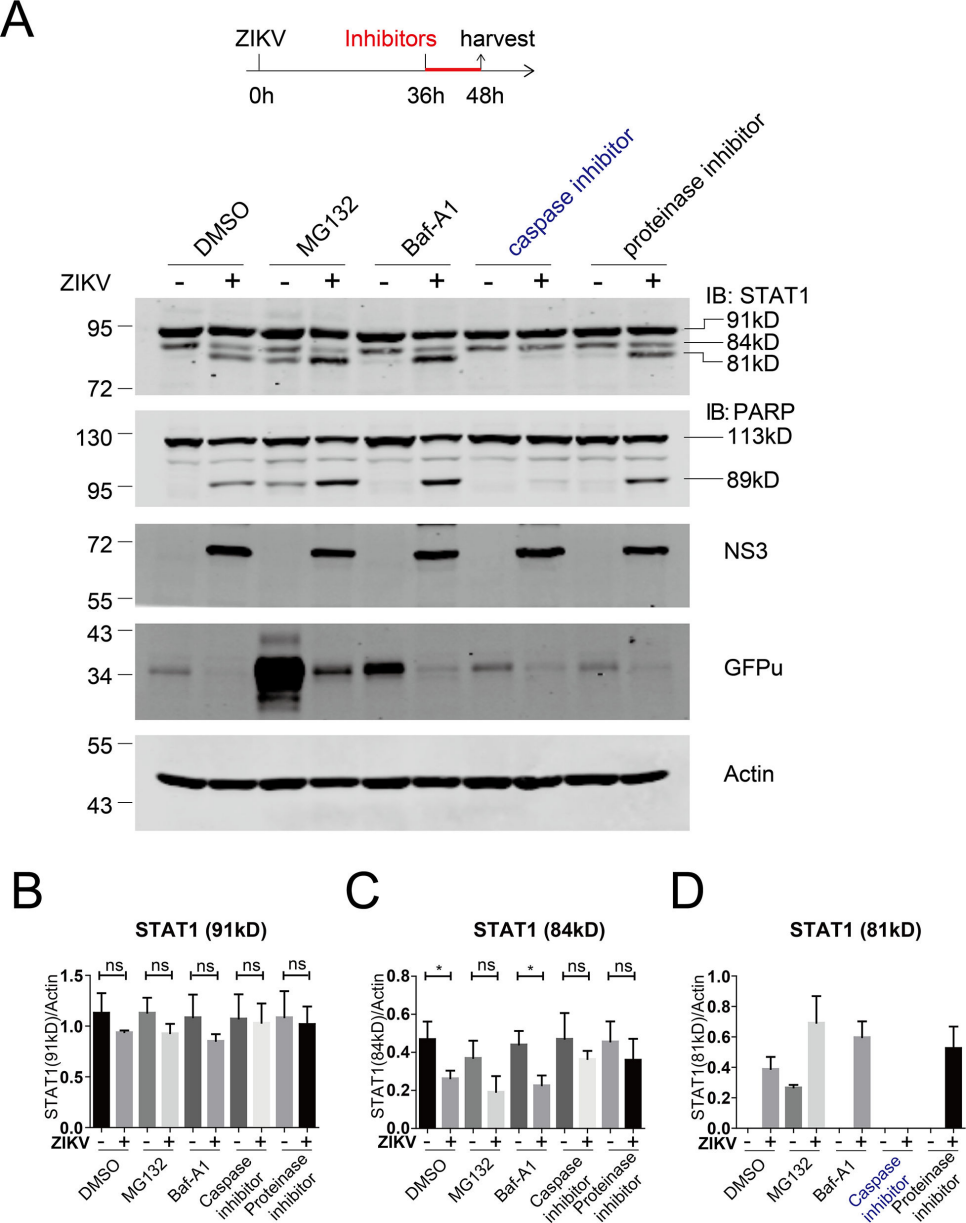
ZIKV infection induced caspase-dependent cleavage of STAT1. (**A**) The upper panel shows the experimental design. Huh7.5-GFPu cells were infected with ZIKV (MOI = 1) for 36 h and then treated with DMSO, MG132 (10 µM), Baf-A1 (0.1 µM), caspase inhibitor (50 µM), or protease inhibitor (1:200) for 12 h and then analyzed by western blotting with the indicated antibodies. Representative pictures of three biological replicates are shown. The values to the left of the blots are molecular sizes in kilodaltons. (**B–D**) The protein abundances of each protein in panel A were quantified and plotted. The mean ± SD of three biological replicates is shown (*n* = 3). Statistical analysis was performed between ZIKV-infected groups and uninfected groups (ns, not significant; *P < 0.05; **P < 0.01; and ***P < 0.001; two-tailed, unpaired *t*-test).

We speculated that the 81 kDa STAT1 is a proteolytic form of the 91 kDa STAT1 and the 84 kDa STAT1. We tried to restore STAT1 cleavage by treating the ZIKV-infected Huh7.5-GFPu cells with various inhibitors, including lysosome inhibitor Baf-A1, caspase inhibitor Z-VAD (OMe)-FMK, and proteinase inhibitor cocktail. At 36 h post-infection, the infected cells were treated with the inhibitors for another 12 h, and the expression of STAT1 was examined. Only the caspase inhibitor prevented the appearance of the 81 kDa STAT1 in the infected cells (Fig. 4A and D). None of these inhibitors affected the degradation of STAT2 (data not shown). Notably, viral infection triggered cleavage of the apoptotic protein poly (ADP-ribose) polymerase (PARP) to form a cleaved 89 kDa species, an indicator of apoptosis (31). The caspase inhibitor also prevented the cleavage of PARP (Fig. 4A). These data indicate that ZIKV infection triggers apoptotic signaling and induces STAT1 cleavage by caspase. Notably, in the uninfected cells, MG132 treatment resulted in a visible STAT1 cleavage (Fig. 4A and D), probably due to the cytotoxicity of MG132 treatment.

### STAT1 was cleaved at residue D694 in ZIKV-infected cells

**Fig 5:**
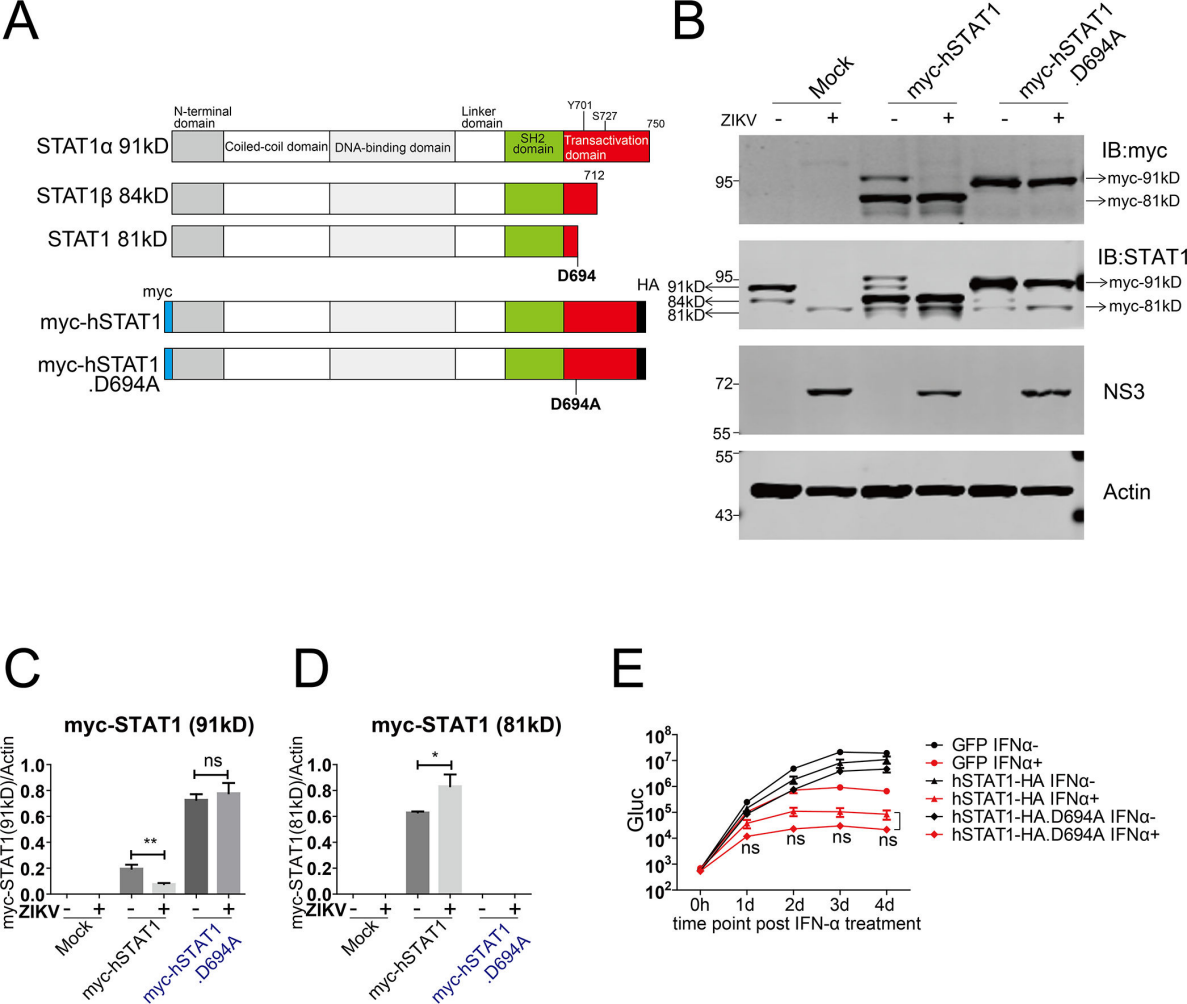
STAT1 was cleaved at residue D694 in ZIKV-infected cells. (**A**) Schematic of STAT1 variants and constructs of STAT1 variants. Phosphorylation sites (Y701 and S727) and the putative caspase cleavage site (D694) are indicated. The HA tag (black bar) was fused in-frame to the C-terminus, and the myc tag (blue) was fused in-frame to the N-terminus of STAT1. (**H–J**) Vero cells were transfected with plasmids expressing myc-STAT1 and myc-STAT1.D694A, and 24 h later, they were infected with ZIKV (MOI = 5) for 4 days. (**B**) The cells were analyzed by western blotting with the indicated antibodies. STAT1 species are indicated (arrows). Representative pictures of three biological replicates are shown. The values to the left of the blots are molecular sizes in kilodaltons. (**C and D**) The protein abundances of each protein in H were quantified and plotted. The mean ± SD of three biological replicates is shown (*n* = 3). Statistical analysis was performed between indicated pairs (ns, not significant; *P < 0.05; **P < 0.01; and ***P < 0.001; two-tailed, unpaired *t*-test). (**E**) Huh7.5-GFP, Huh7.5-hSTAT1-HA, and Huh7.5-hSTAT1-HA.D694A were infected with C7-Gluc (MOI = 0.4) for 8 h and then treated with IFN-α (2,000 U/mL). At the indicated time points after IFN-α treatment, cells were harvested. Gluc activities in the cell lysates were determined. The mean ± SD of six biological replicates is shown (*n* = 6). Statistical analysis was performed between the indicated groups at each time point (ns, not significant; two-way ANOVA).

STAT1 has two isoforms (α and β) formed by alternative splicing. STAT1α and STAT1β share most amino acids but have different C-termini. STAT1α represents the 91 kDa species, and STAT1β represents the 84 kDa species with a shortened C-terminus (Fig. 5A) (32). It has been reported that in Hela cell, double-stranded RNA (dsRNA) supplemented in the growth media induces apoptosis to cleave STAT1 at aspartic acid 694 by caspase, forming an 81 kDa STAT1 (33), which is similar to our observation. We speculated that the 81 kDa STAT1 in the ZIKV-infected cells is the STAT1 cleaved at aspartic acid 694. We constructed lentiviral plasmids to express C-terminal HA-tagged STAT1α and STAT1α with the point mutation D694A (Fig. 5A). We generated Huh7.5 cell lines stably expressing STAT1α (human STAT1, hSTAT1) and STAT1α.D694A (hSTAT1.D694A), respectively (Fig. 5A). In the Huh7.5-GFP control cell lines, ZIKV infection at an MOI of 5 induced cleavage of the endogenous STAT1 and the accumulation of the 81 kDa STAT1. In the Huh7.5-hSTAT1-HA cell lines, the exogenous hSTAT1-HA was readily cleaved in the ZIKV-infected cells, as evidenced by a significant reduction of the STAT1-HA protein levels. In contrast, in the Huh7.5-hSTAT1-HA.D694A cell lines, no significant reduction of the STAT1-HA.D694A protein was observed (data not shown). We could not detect the cleaved products of the HA-tagged STAT1 because the HA tag was fused to the C-terminus of STAT1 (Fig. 5A).

To further verify the cleavage of STAT1, we constructed plasmids to express N-terminal myc-tagged hSTAT1α-HA and hSTAT1α-HA.D694A, respectively (Fig. 5A). We transfected the plasmids into Vero cells and then infected the cells with ZIKV to observe the cleavage of the transfected STAT1 variants. Overexpression of myc-STAT1 in the mock-infected cells resulted in the production of a shorter STAT1, corresponding to the expected cleaved 81-kDa STAT1. In the ZIKV-infected cells, the 91-kDa full-length STAT1 (myc-STAT1) disappeared, but the 81-kDa cleaved STAT1 remained consistent (Fig. 5B through D). In contrast, overexpression of myc-STAT1.D694A did not result in the production of the 81-kDa STAT1, either in the mock-infected or in the ZIKV-infected cells, suggesting that the myc-STAT1.D694A was resistant to cleavage in the ZIKV-infected cells (Fig. 5B through D). Taken together, these data demonstrate that STAT1 is cleaved at D694 in ZIKV-infected cells.

To explore the physiological relevance of STAT1 cleavage at D694 in viral IFN antagonism, we sought to examine if the cleavage-resistant mutant STAT1.D694A could exert stronger antiviral activity than the cleavable wild-type STAT1 upon IFN treatment. We infected Huh7.5-GFP, Huh7.5-hSTAT1-HA, and Huh7.5-hSTAT1.D694A cells with ZIKV-Gluc at an MOI of 0.4. Then, we treated the cells with or without IFN-α and assessed the viral replication by measuring Gluc activity (Fig. 5E). In the hSTAT1α.D694A cells, IFN elicited a comparable antiviral activity with that in the hSTAT1α cell (Fig. 5E), suggesting that resistance of hSTAT1 to cleavage does not render stronger antiviral activity.

### Murine STAT1 restricted ZIKV infection in murine cell

**Fig 6:**
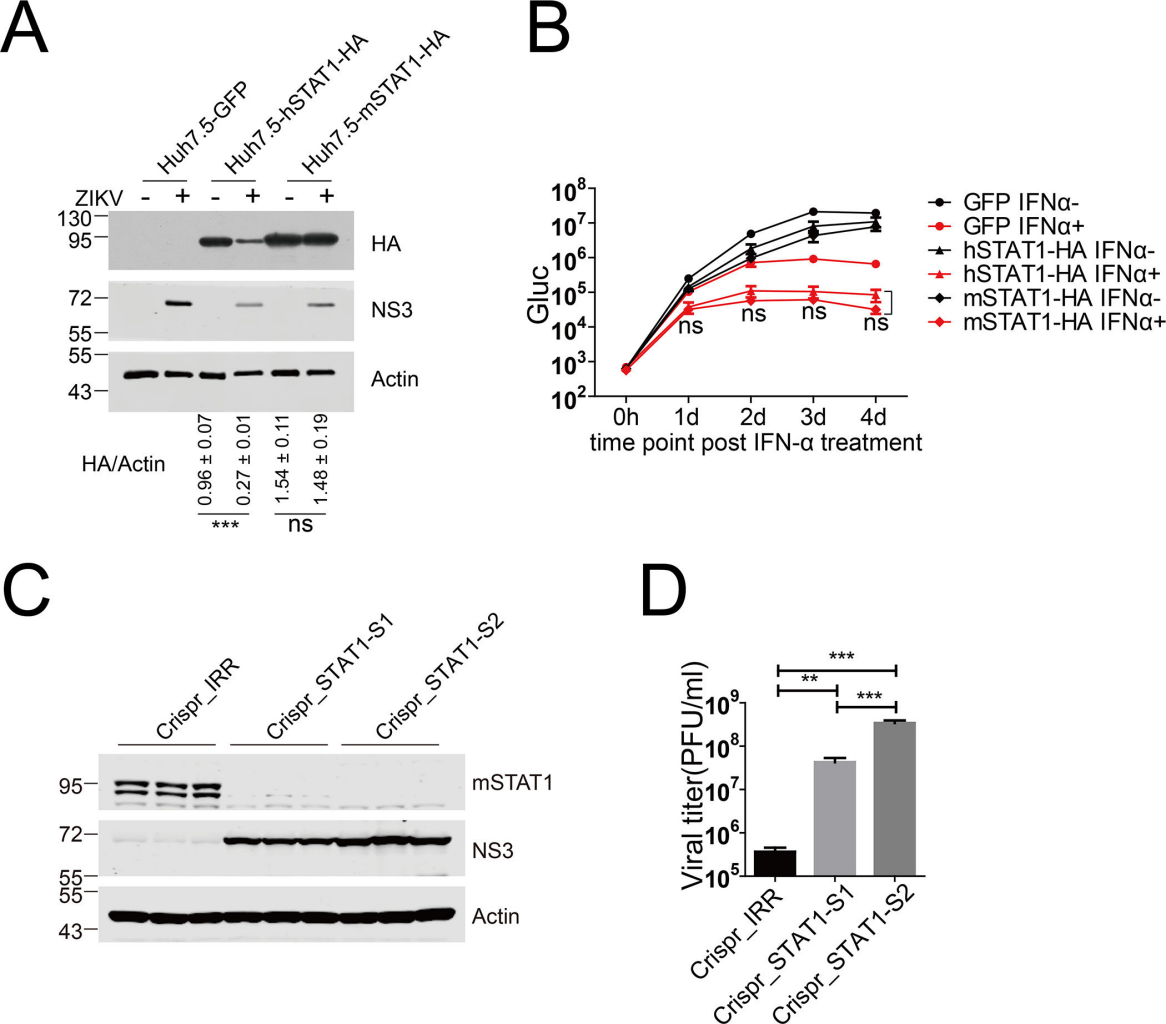
Murine STAT1 restricted ZIKV infection in murine cells. (**A**) Huh7.5 cells stably expressing GFP, hSTAT1-HA, and murine STAT1-HA (mSTAT1-HA) were infected with ZIKV (MOI = 5) for 4 days and then harvested for western blotting analysis with the indicated antibodies. Representative pictures of three biological replicates are shown. The values to the left of the blots are molecular sizes in kilodaltons. (**B**) Huh7.5-GFP, Huh7.5-hSTAT1-HA, and Huh7.5-mSTAT1-HA cells were infected with ZIKV-Gluc (MOI = 0.4) for 8 h and then treated with IFN-α (2,000 U/mL). At the indicated time points after IFN-α treatment, cells were harvested. Gluc activities in the cell lysates were determined. The mean ± SD of six biological replicates is shown (*n* = 6). Statistical analysis was performed between the indicated groups at each time point (ns, not significant; two-way ANOVA). (**C**) NIH-3T3 Crispr_IRR, Crispr_STAT1-S1, and Crispr_STAT1-S2 were infected with ZIKV (MOI = 5) for 4 days and then analyzed by western blotting and plaque assay. Western blot analysis of infected cells with the indicated antibodies. Representative pictures of three biological replicates are shown. The values to the left of the blots are molecular sizes in kilodaltons. (**D**) Virus titers in the supernatants of panel **C** were quantified and plotted. The mean ± SD of three biological replicates is shown (*n* = 3). Statistical analysis was performed between ZIKV-infected groups and uninfected groups (ns, not significant; *P < 0.05; **P < 0.01; and ***P < 0.001; two-tailed, unpaired *t*-test).

Previous studies have shown that ZIKV and DENV NS5 bind and degrade hSTAT2 but not murine STAT2 (mSTAT2), which may contribute to host tropism (23, 34). First, we explored if ZIKV infection induces cleavage of mSTAT1. We constructed lentiviral plasmids to express murine STAT1 with an HA tag on the C termini similarly as hSTAT1 described above and then generated Huh7.5 cell lines stably expressing mSTAT1. We infected Huh7.5-hSTAT1 and Huh7.5-mSTAT1 with ZIKV at an MOI of 5. As described above, hSTAT1 was readily cleaved in the infected cells, as evidenced by a reduction in the protein level of STAT1-HA, whereas the protein level of mSTAT1 was not significantly affected upon viral infection (Fig. 6A). We then assessed if the cleavage-resistant mSTAT1 could elicit stronger antiviral activity upon IFN treatment. We infected Huh7.5-GFP, Huh7.5-hSTAT1-HA, and Huh7.5-mSTAT1-HA cells with ZIKV-Gluc at an MOI of 0.4. Then, we treated the cells with or without IFN-α and assessed viral replication by measuring Gluc activity. Murine STAT1 exhibited similar antiviral activity as hSTAT1 upon IFN treatment (Fig. 6B).

To further investigate the role of mSTAT1 in restricting ZIKV infection, we knocked out mSTAT1 in mouse embryonic fibroblast NIH3T3 cells by CRISPR. We generated CRISPR-mediated STAT1 knockout cell lines and selected two colonies (S1 and S2) by single cell growing. Knockout efficiency was verified by Sanger sequencing the amplicon of a sgRNA-targeted region (data not shown). Then, the S1 and S2 cells were infected with ZIKV with an MOI of 5, and the results showed that the expression of viral protein NS3 in these mSTAT1 knockout cells was significantly increased compared with the control cells (Crispr_IRR) (Fig. 6C). In addition, we titrated the virus in the supernatant of the infected cells, and there was significantly higher virus titer in the mSTAT1 knockout cells than in the control cells (Fig. 6D). These data indicate that STAT1 signaling is essential for restricting ZIKV infection in murine cell.

### Determinants of ZIKV infection-induced murine STAT1 cleavage

**Fig 7:**
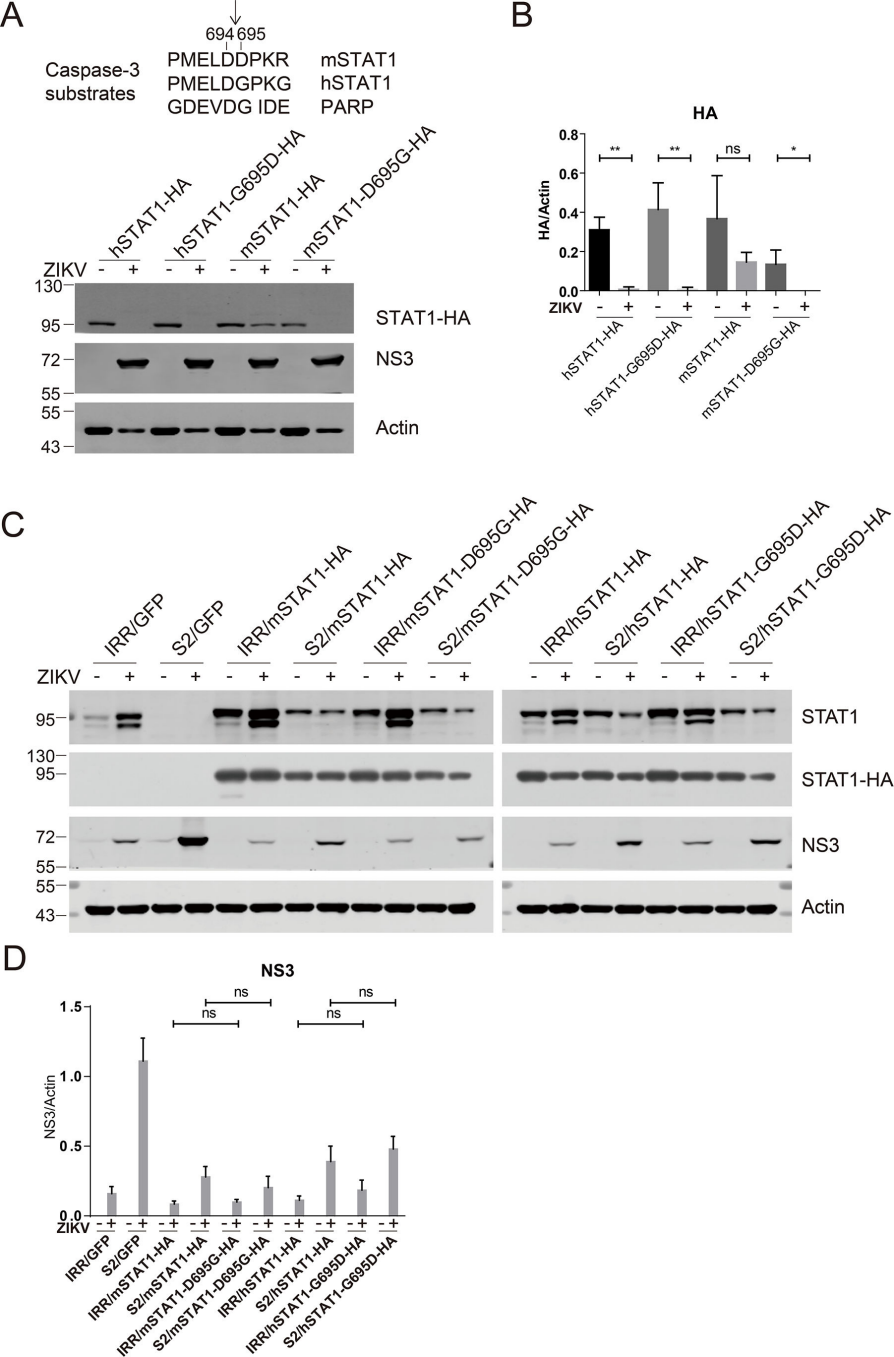
Determinants of murine STAT1 cleavage and complementation of murine STAT1 variants in mSTAT1 knockout cells. (**A**) The upper panel shows partial sequences of murine STAT1, human STAT1, and the known caspase substrate PARP. The cleavage sites of human STAT1 (after aspartic acid 694) and PARP are indicated with arrows. Vero cells were transfected with plasmids expressing hSTAT1-HA, hSTAT1-G695D-HA, mSTAT1-HA, and mSTAT1-D695G-HA for 24 h, and then infected with ZIKV (MOI = 5) for 4 days. The cells were analyzed by western blotting with the indicated antibodies. STAT1 species are indicated. Representative pictures of three biological replicates are shown. The values to the left of the blots are molecular sizes in kilodaltons. (**B**) The protein abundances of each protein in panel A were quantified and plotted. The mean ± SD of three biological replicates is shown (*n* = 3). Statistical analysis was performed between indicated pairs (ns, not significant; *P < 0.05; **P < 0.01; and ***P < 0.001; two-tailed, unpaired *t*-test). (**C**) NIH-3T3 Crispr_IRR and Crispr_STAT1-S2 cells stably expressing GFP, mSTAT1-HA, mSTAT1-D695G-HA, hSTAT1-HA, and hSTAT1-G695D-HA were infected with ZIKV (MOI = 5) for 4 days and then analyzed by western blotting with the indicated antibodies. Representative pictures of three biological replicates are shown. The values to the left of the blots are molecular sizes in kilodaltons. (**D**) The protein abundances of protein bands in panel A were quantified and plotted. The mean ± SD of three biological replicates is shown (*n* = 3). Statistical analysis was performed between ZIKV-infected groups and uninfected groups (ns, not significant and *P < 0.05; two-tailed, unpaired *t*-test).

As murine STAT1 is resistant while human STAT1 is sensitive to ZIKV infection-induced cleavage, we compared the amino acid sequence of human STAT1 and murine STAT1 and found that the amino acid at 694 and 695 positions of human STAT1 is DG (aspartic acid and glycine), while the amino acid at the corresponding position of murine STAT1 is DD (aspartic acid and aspartic acid). We speculated that the difference in the amino acid at position 695 may contribute to the resistance of mSTAT1 to ZIKV infection-induced cleavage. We constructed the plasmid to express a C-terminal HA-tagged human STAT1α with the glycine residue at position 695 changed to aspartic acid (hSTAT1-G695D-HA) and the plasmid to express a C-terminal HA-tagged murine STAT1α with the aspartic acid residue at position 695 changed to glycine (mSTAT1-D695G-HA). Vero cells were transfected with wild-type human and murine hSTAT1 and their mutants for 24 h and then infected with ZIKV for 4 days. The expression of the HA-tagged STAT1 variants was analyzed by western blotting. The wild-type human STAT1 (hSTAT1) and human STAT1-G695D were readily cleaved in the ZIKV-infected cells (Fig. 7A and B). Wild-type murine STAT1 (mSTAT1), as expected, was resistant to ZIKV infection-induced cleavage. In contrast, mSTAT1-D695G was readily cleaved in the ZIKV-infected cells, suggesting that D695 is the determinant amino acid for murine STAT1 cleavage upon ZIKV infection (Fig. 7A and B).

### Complementation of murine STAT1 variants in mSTAT1 knockout cells and their effects on antiviral signaling

To further investigate the physiological relevance of the STAT1 cleavage in antiviral signaling antagonism, we carried out a complementation experiment in STAT1 knockout mouse embryonic fibroblast NIH3T3 cells. We stably overexpressed GFP, mSTAT1-HA, mSTAT1-D695G-HA, hSTAT1-HA, and hSTAT1-G695D-HA in the NIH3T3 Crispr_IRR cells and the NIH3T3 Crispr_STAT1-S2 cells. Then, these cells were infected with ZIKV with an MOI of 5. The antiviral signaling was assessed by quantitation of viral NS3 protein by western blotting. Compared with Crispr_IRR cells, in the Crispr_STAT1-S2 cells, ZIKV infection was significantly increased, as evidenced by elevated NS3 expression (Fig. 7C and D). In the Crispr_STAT1-S2 cells, complementation of mSTAT1 resulted in a significant reduction of the NS3 protein levels, compared with complementation of GFP (Fig. 7C and D), suggesting that the complemented mSTAT1 elicited efficient antiviral signaling. Intriguingly, complementation of the mSTAT1-D695G that is supposed to be efficiently cleaved during ZIKV infection also resulted in a significant reduction of ZIKV NS3 protein levels, compared with complementation of GFP (Fig. 7C and D). We also complemented hSTAT1 and hSTAT1-G695D in the Crispr_IRR cells and Crispr_STAT1-S2 cells, respectively. Similarly, expression of these STAT1 variants resulted in a significant reduction of ZIKV NS3 protein levels (Fig. 7C and D). Taken together, these data demonstrate that complementation of murine cleavable STAT1 variant elicits comparable antiviral signaling as the cleave-resistant murine wild-type STAT1, which suggests that ZIKV infection-induced STAT1 cleavage is dispensable for antiviral evasion in murine cells.

## DISCUSSION

It has been well-documented that flaviviruses employ either NS5-dependent or NS5-independent mechanisms to antagonize STAT2-mediated IFN signaling (20 – 23). By using infectious clones of ZIKV MR766, we recently demonstrated that ZIKV infection triggers the suppression of host *de novo* protein synthesis to accelerate the degradation of short-lived, ubiquitinated STAT2 (24). Herein, we extended our study to explore the effect of ZIKV infection on STAT1. The effect of ZIKV infection on STAT1 is controversial. Several studies reported that ZIKV does not affect STAT1 expression (22, 23), while other studies demonstrate that ZIKV infection reduces the phosphorylation of STAT1 and STAT2 (28, 29). NS5 of JEV has been reported to interfere with the phosphorylation of Tyk2 and STAT1 and inhibit the nuclear translocation of STAT1 (26). TBEV NS4A blocks the phosphorylation of STAT1 and STAT2 and the dimerization of STAT1 and STAT2 (27).

In this study, we first found that ZIKV infection did not affect STAT1 phosphorylation (Fig. 1B and D). During late infection, STAT1 was cleaved at aspartic acid 694 in a caspase-dependent manner (Fig. 2 to 5). It has been reported that dsRNA induces apoptosis to cleave STAT1 at amino acid 694 by caspase (33). It is reasonable that during late infection, accumulation of dsRNA generated by viral replication triggers apoptosis to cleave STAT1. It also should be noted that, like other positive-stranded RNA viruses, the viral replication and the dsRNA products of flaviviruses may be shielded by double-membranous vesicles (35), which makes the dsRNA inaccessible. Alternatively, in the ZIKV-infected cells, other viral replication-induced signals trigger apoptosis to cleave STAT1.

Murine STAT1 was resistant to ZIKV infection-induced cleavage (Fig. 6). The amino acid G695 is the determinant of mSTAT1 cleavage in ZIKV-infected cells and the G695D mutation renders mSTAT1 sensitive to ZIKV infection-induced cleavage (Fig. 7A and B). There are two phosphorylated sites (tyrosine 701 and 727) on the C-terminus of STAT1, which are important for STAT1 nuclear translocation (36, 37). Cleavage at D694 results in C-terminal truncation, which inactivates STAT1. The mouse is resistant to ZIKV infection, whereas STAT1 knockout mouse is highly sensitive to ZIKV (25), suggesting that STAT1 signaling plays an important role in restricting ZIKV infection. STAT1 knockout in mouse embryonic fibroblast NIH3T3 cells dramatically augmented ZIKV infection (Fig. 6C and D). These data prompted us to speculate that STAT1 cleavage is a mechanism of IFN antagonism, and the resistance of murine STAT1 to cleavage may contribute to viral restriction in mice.

To explore the physiological relevance of STAT1 cleavage in IFN antagonism, we first assessed the antiviral signaling in the hSTAT1 and the cleavage-resistant hSTAT1.D694A stably expressed cell lines (Fig. 5B and D). We compared the antiviral activity of IFN treatment in these cells. Intriguingly, hSTAT1α.D694A exhibited comparable antiviral activity with hSTAT1α upon IFN treatment (Fig. 5E). Furthermore, we performed a complementation experiment in a STAT1 knockout NIH3T3 cell colony by stably expressing mSTAT1 and the cleavable mSTAT1.D695G. Complementation of both the mSTAT1 and the mSTAT1.D695G rescued robust antiviral activity in the STAT1 knockout cells (Fig. 7C and D). These data argue for the dispensable role of ZIKV infection-induced STAT1 cleavage in IFN antagonism during viral infection. These observations may be explained by the fact that STAT1 cleavage takes place during late infection when the viral infection has been established. These data may also imply the dominant role of antagonism of STAT2 but not STAT1 in ZIKV-infected cells and further efforts might be focused on dissecting the mechanisms of STAT2 antagonism.

However, we could not completely exclude the physiological relevance of STAT1 cleavage in viral evasion of host innate immunity. It has been reported that in ZIKV-infected human macrophages, STAT1 acts as a key transcription factor for the induction of CH25H (38), a molecular mediator of innate antiviral immunity against both enveloped and non-enveloped viruses (39 – 44). It is worthy to note that STAT1 participates in type I, type II, and type III IFN signaling (45). How the cleavage of STAT1 by ZIKV blocks type I, type II, and type III IFN signaling needs further study.

## MATERIALS AND METHODS

### Plasmids

To generate the plasmid pTrip-IRES-puro-GFP, the GFP coding sequence was cloned into the XbaI/BamHI site in a homemade plasmid pTrip-IRES-puro. To generate the plasmids pTRIP-IRES-puro-mSTAT1-HA, coding regions of murine STAT1 were amplified from a cDNA clone (Mouse Tagged ORF Clone MR227437, OriGene), flanking the HA tag in the C-termini of the ORFs, and then cloned into the XhoI/BamHI site in the plasmid pTrip-IRES-puro, respectively. To generate the plasmid pTRIP-IRES-puro-STAT1-HA, the coding sequence for human STAT1 and a C-terminally flanked HA tag was synthesized and cloned into the BsrGI/BamHI site in the plasmid pTRIP-IRES-puro. The D694A mutation was introduced into the pTrip-IRES-puro-hSTAT1-HA by fusion PCR mediated-mutagenesis to get the plasmid pTrip-IRES-puro-hSTAT1-HA.D694A. A myc tag was added into the N-terminal STAT1 in the plasmids pTrip-IRES-puro-hSTAT1-HA and pTrip-IRES-puro-hSTAT1-HA.D694A to get the plasmids pTrip-IRES-puro-myc-hSTAT1-HA and pTrip-IRES-puro-myc-hSTAT1-HA.D694A, respectively. The G695D mutation was introduced into the pTrip-IRES-puro-hSTAT1-HA by fusion PCR mediated-mutagenesis to get the plasmid pTrip-IRES-puro-hSTAT1-G695D.HA. The D695G mutation was introduced into the pTrip-IRES-puro-mSTAT1-HA by fusion PCR mediated-mutagenesis to get the plasmid pTrip-IRES-puro-mSTAT1-D695G.HA.

For CRISPR-mediated knockdown (Kd), CRISPR single guide RNA (sgRNA)-mediated gene Kd oligos encoding subgenomic RNAs against an irrelevant target (IRR) (5′-caccgATA GCG ACT AAA CAC ATC AA-3′), the oligonucleotides targeting mouse STAT1 (46, 47), sg1 (5′-caccgGGA TAG ACG CCC AGC CAC TG-3′), sg2 (5′-caccgTGT GAT GTT AGA TAA ACA GA-3′), sg3 (5′-caccgTTA ATG ACG AGC TCG TGG AG-3′), sg4 (5′-caccgGAA AAG CAA GCG TAA TCT CC-3′), sg5 (5′-caccgGGT CGC AAA CGA GAC ATC AT-3′), sg6 (5′-caccgACT GGT CGT CCA GCT GTG AG-3′), and sg7 (5′-caccgGAT CAT CTA CAA CTG TCT GA-3′) were synthesized and ligated into LentiCRISPR-V2 (Addgene plasmid no. 52961).

### Cell lines

Huh7.5 cell lines (kindly provided by Charles Rice) were routinely maintained in Dulbecco’s modified Eagle’s medium (DMEM) containing 10% fetal bovine serum (FBS) (Biological Industries catalog no. 04-001-1), 25 mM HEPES (Gibco), and nonessential amino acids (Gibco). African Green Monkey Kidney (Vero) cells (Cell Bank of the Chinese Academy of Sciences, Shanghai, China) were routinely maintained in a similar medium supplemented with 1% penicillin. HEK-293T and NIH-3T3 cell lines were purchased from the National Collection of Authenticated Cell Cultures, Shanghai, China (www.cellbank.org.cn). HEK-293T and NIH-3T3 were cultured in Dulbecco’s modified Eagles’s medium supplemented with 10% FBS (Gibco), 25 mM HEPES (Gibco), and 1% penicillin/streptomycin (Biological Industries).

Huh7.5 cell lines overexpressing Trip-IRES-puro-GFP, pTrip-IRES-puro-hSTAT1-HA, pTrip-IRES-puro-mSTAT1-HA, and pTrip-IRES-puro-hSTAT1-HA.D694A were generated by transducing Huh7.5 cells with vesicular stomatitis virus G protein (VSV-G)-pseudotyped lentiviral particles and growing cells in a conditioned medium supplemented with 5 µg/mL puromycin. The surviving cells were pooled and maintained in a conditioned medium with 0.5 µg/mL puromicin. For CRISPR-mediated knockdown of STAT1, NIH-3T3 cell lines were transduced with VSV-G-pseudotyped LentiCRISPR lentiviral particles and selected with a conditioned medium supplemented with 5 µg/mL puromicin. The surviving cells were pooled and maintained in a conditioned medium supplemented with 5 µg/mL puromicin.

For single-cell isolation of STAT1 knockout cell, we sorted a pooled cell line of Crispr-STAT1 (sg6) using a limited dilution method. The single-cell growing colonies were first identified by western blotting analysis with an anti-murine STAT1 antibody. The colonies were further identified by harvesting genomic DNA. A region encompassing the sgRNA targeted sequence was amplified by PCR and sequenced by Sanger sequencing.

### Antibodies and chemicals

Anti-STAT1 antibody (9172; Cell Signaling Technology) and anti-pSTAT1 (9167; Cell Signaling Technology) antibody were used at 1:1,000 dilution. Anti-PARP (A19596; ABclonal) was used at a 1:1,000 dilution for western blotting. Anti-ZIKV NS5 antibody (GTX133312; Genetex), anti-ZIKV NS3 antibody (GTX133309; Genetex), and anti-YFV NS5 antibody (GTX134141; Genetex) were used in western blots at 1:1,000 dilution in immunostaining. An anti-β-Actin antibody (A1978; Sigma) was used at a 1:4,000 dilution. Anti-HA antibody from Roche (11867423001) was used at 1:500 dilution. An anti-GFP antibody from Santa Cruz Biotechnology (sc-9996) was utilized at a 1:1,000 dilution. Goat-anti-mouse IRDye 800CW secondary antibody (LI-COR; 926-32210) was used at 1:10,000 dilution; goat-anti-rabbit IRDye 800 CW secondary antibody (LI-COR; 926-32211) was used at 1:10,000 dilution; goat-anti-mouse HRP IgG (Santa Cruz; sc-2005) was used at 1:2,000 dilution; goat-anti-rat HRP IgG (Santa Cruz; sc-2006) was used at 1:2,000 dilution; and goat-anti-rabbit HRP IgG (Santa Cruz; sc-2004) was used at 1:2,000 dilution.

Bafilomycin A1 (B1793; Sigma) was used at a final concentration of 0.1 µM. Z-VAD (OMe)-FMK (HY-16658; MCE) was used at a final concentration of 50 µM. A protease inhibitor cocktail (P1860, Sigma) was used at a final concentration of 1:200. MG132 (M7449; Sigma) was used at a final concentration of 10 µM. IFN-α was purchased from PBL Assay Science (11200-2).

### Virus

ZIKV MR766 (C7) was generated by electroporation of *in vitro*-transcribed viral RNAs into Vero cells as described previously (24), and the virus titer was determined in Vero cells by plaque assay. VSV-G-pseudotyped lentiviral particles were generated by co-transfection of HEK293T cells with plasmids encoding VSV-G and HIV gag-pol and with the lentiviral provirus plasmids. The medium overlying the cells was harvested at 48–72 h after transfection, filtered through a 0.45 µm filter, and stored at −80°C. Cells were transduced with the pseudoparticles in the presence of 8 µg/mL Polybrene.

### Plaque assay

ZIKV was titrated by infection of Vero cells with 10-fold serial dilutions in DMEM with 2% fetal calf serum(FCS). A total of 200 µL of diluted virus was added to each well in a 6-well plate and after 1 h of infection, the well was overlaid with 0.6% agarose in MEM supplemented with 2% FBS. Plaques were enumerated by crystal violet staining after 7 days for ZIKV.

### Luciferase activity

The cells in the 48-well plate were lysed in 60 µL of 1× passive lysis buffer (Promega). Luciferase activity was measured with Renilla luciferase substrate (Promega) according to the manufacturer’s protocol.

### Western blotting

After washing with phosphate buffered saline (PBS) , cells were lysed with 2× SDS loading buffer [100 mM Tris-Cl (pH 6.8), 4% SDS, 0.2% bromophenol blue, 20% glycerol, and 10% 2-mercaptoethanol] and then boiled for 10 min. Proteins were separated by SDS-PAGE and transferred to a nitrocellulose membrane. The membranes were incubated with blocking buffer (PBS, 5% milk, and 0.05% Tween) for 1 h and then with primary antibody diluted in the blocking buffer. After three washes with PBST (PBS, 0.05% Tween), the membranes were incubated with a secondary antibody. After three washes with PBST, the membrane was visualized by Western Lightning Plus-ECL substrate (PerkinElmer; NEL10500) or by an Odyssey CLx Imaging System. The protein bands were quantified by densitometry with ImageJ if necessary.

### Statistical analysis

Statistical analysis was performed with the GraphPad Prism 8 software. Specific tests are described in the figure legends.
